# In Ovo CAM-Based Xenograft Model for Investigating Tumor Developmental Biology in Breast Cancer

**DOI:** 10.21769/BioProtoc.5600

**Published:** 2026-02-20

**Authors:** Carlos César Patiño Morales, Claudia Haydée González de La Rosa, Ricardo Jaime-Cruz, Marcela Salazar-García, Laura Villavicencio Guzmán, Ana Karen Herrera-Vargas

**Affiliations:** 1 Departamento de Ciencias Naturales, Unidad Cuajimalpa, Universidad Autónoma Metropolitana, Ciudad de México, México; 2 Laboratorio de Investigación en Biología del Desarrollo y Teratogénesis Experimental/Hospital Infantil de México Federico Gómez, Mexico City, México; 3 Laboratorio de Biología Celular del Cáncer/ Universidad Autónoma de Guerrero, Chilpancingo, Guerrero, México

**Keywords:** Breast cancer, CAM assay, Xenograft, In ovo model, Chick embryo

## Abstract

Breast cancer remains one of the most prevalent and deadly malignancies affecting women worldwide. Its progression and metastatic behavior are driven by complex mechanisms. To develop more effective therapeutic strategies, it is crucial to understand tumor growth, angiogenesis, and microenvironmental interactions. Although traditional in vivo models such as murine xenografts have long been used to study tumor biology, these approaches are often time-consuming, costly, and ethically constrained. In contrast, the chick embryo chorioallantoic membrane (CAM) assay offers a rapid, cost-effective, and ethically flexible alternative for evaluating tumor development and angiogenesis. This protocol describes an in ovo CAM-based xenograft model in which human breast cancer cells are implanted onto the vascularized CAM of chick embryos. This method enables real-time evaluation of tumor growth. Furthermore, the model allows for manipulation of experimental conditions, including pharmacological treatments or genetic modifications, to study specific molecular mechanisms involved in breast cancer progression. The major advantages of this protocol lie in its simplicity, reduced cost, and capacity for high-throughput screening, making it a valuable tool for translational cancer research.

Key features

• Enables rapid tumor formation (3–4 days) after implantation of breast cancer cells.

• Ethical and low-cost alternative to rodent xenograft models; suitable for laboratories without animal facility infrastructure and aligned with the 3Rs principles.

• Optimized for short-term studies of tumor development, angiogenesis, and early metastatic events.

• Highly suitable for pharmacological testing and experimental manipulation of the tumor microenvironment.

## Graphical overview


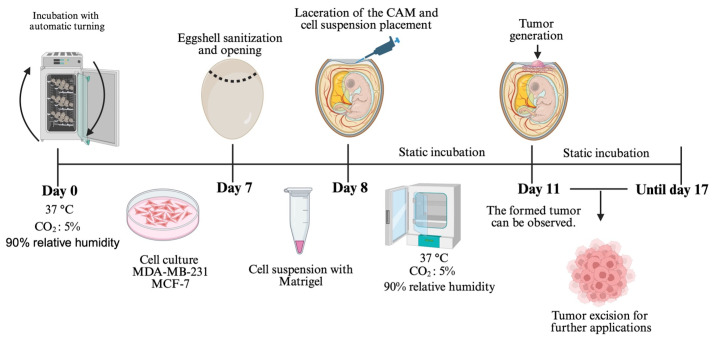


**Chorioallantoic membrane (CAM) xenograft protocol.** Fertilized eggs are incubated under automatic turning conditions until day 7, when eggshell windowing is performed. On day 8, the cell–Matrigel suspension is placed onto the exposed CAM. Embryos are maintained under static incubation, and tumor formation becomes visible from day 11. Tumors are collected for downstream applications.

## Background

Breast cancer remains a significant global health concern. It is the most commonly diagnosed cancer in women and a leading cause of cancer-related deaths worldwide [1,2]. Epidemiological data indicate a continuous rise in incidence, largely associated with lifestyle factors, aging populations, and increased screening [3]. Despite substantial progress in clinical management driven by molecular subtyping, targeted therapies, immunotherapy, and improvements in early detection in recent decades, the clinical outcome for many patients continues to be negatively influenced by high tumor heterogeneity and frequent therapeutic resistance acquisition [4]. These characteristics limit the long-term success of current treatments and highlight the necessity of experimental approaches that allow a more precise understanding of breast cancer biology [2]. To achieve this, research models must not only replicate tumor-intrinsic molecular features but also key components of the tumor microenvironment, including stromal interactions, extracellular matrix composition, angiogenic processes, and the dynamic exchange of soluble factors [5]. Conventional two-dimensional (2D) cell culture systems have played an essential role in elucidating molecular pathways associated with proliferation, migration, and survival; however, they fail to reproduce the architectural and biochemical complexity of tumors in vivo [6]. Recently, three-dimensional (3D) culture platforms, including spheroids, scaffolds, organoids, and bioprinted constructs, have improved the modeling of cell–cell and cell–matrix interactions. However, limitations still exist when using these platforms to study angiogenesis, invasion, immune interactions, and metastatic potential [7]. For this reason, animal models are often necessary to validate in vitro observations and evaluate therapeutic responses in a context that is physiologically relevant [8].

Among animal systems, murine xenograft models have historically been the gold standard in preclinical breast cancer research. They permit long-term tumor development and allow evaluation of metastasis, immune modulation, and therapeutic efficacy. Nonetheless, their routine use poses significant challenges [9]. Rodent studies require specialized facilities, rigorous ethical reviews, significant financial resources, and extended timeframes for experimentation. These challenges, coupled with the growing emphasis on reducing, refining, and replacing animal experimentation (the 3Rs), have driven the development of alternative models that minimize animal suffering while maintaining biological relevance [10,11].

In this context, the chorioallantoic membrane (CAM) of the chick embryo has emerged as a powerful, versatile, and ethically advantageous platform for in vivo cancer research [12]. The CAM is a highly vascularized extraembryonic membrane that supports rapid tumor engraftment and neovascularization [13,14]. Importantly, the chicken embryo lacks a fully developed immune system during early incubation, which allows implantation of human cancer cells without the need for immunosuppression [15,16]. This cost-effective model requires minimal infrastructure and enables experimental timelines that are significantly shorter than those observed in rodents. Additionally, the CAM is not innervated during the early stages of development, which minimizes potential pain and reduces ethical concerns [17–21].

The present protocol was developed to provide a detailed, standardized, and reproducible methodology for inducing tumors on the CAM using human breast cancer cell lines. While the model has been successfully applied to melanoma, glioblastoma, colorectal, and head and neck cancers, breast cancer remains one of the most studied tumor types in CAM systems due to its strong angiogenic profile and rapid growth [22]. Here, we outline the entire workflow from cell preparation and embryo windowing to tumor implantation, monitoring, histological processing, and downstream analysis. Using MDA-MB-231 and MCF-7 cells, this protocol enables the formation of measurable tumors within 72 h, allowing researchers to quickly assess tumor morphology and angiogenic characteristics. The model is also compatible with drug testing, gene silencing, overexpression, extracellular vesicle delivery, and co-culture with stromal or immune components [23,24].

Despite its advantages, the CAM system has limitations. Because embryonic development progresses rapidly, experiments must be completed within a limited timeframe, which prevents long-term studies of metastasis or tumor dormancy. Additionally, the immature immune system of the embryo hinders the evaluation of intricate adaptive immune responses. The relatively small size of the embryo and tumors may also restrict surgical manipulation and imaging resolution [25–27]. However, ongoing technical innovations (including intravital microscopy, micro-CT imaging, transcriptomic profiling, and nanoparticle tracking) are expanding the versatility of CAM-based approaches [23,28].

Overall, the CAM model provides an ethical, economical, and biologically relevant alternative for preclinical breast cancer research. Studies have demonstrated that CAM-derived tumors preserve histological architecture, proliferative markers, and angiogenic profiles closely resembling those observed in murine models and human patient specimens [23,29]. Consequently, this platform facilitates rapid, high-throughput in vivo experimentation while significantly reducing the use of mammalian animals [30]. Although the CAM model offers several experimental advantages, it also has intrinsic biological limitations that need to be considered. The experimental timeframe is limited, typically concluding around day 14 of embryonic development, which restricts long-term investigations of tumor growth, metastasis, and delayed therapeutic responses [31]. Furthermore, the chick embryo’s immunologically immature environment does not fully replicate the complex tumor–immune interactions observed in adult organisms, limiting its use for immunotherapy studies [32]. Therefore, the CAM assay should be viewed as a complementary preclinical platform rather than a substitute for established murine models. This protocol provides a practical, highly adaptable tool for studying tumor biology, evaluating therapeutic interventions, and connecting in vitro systems with mammalian models. While the primary focus of this study is breast cancer, the CAM model has also been successfully used to generate tumors from other cancer cell lines in our laboratory. These include neuroblastoma [SH-SY5Y, Be(2)-C], cervical cancer (HeLa, SiHa), lung cancer (H1299), and melanoma (A-375). This demonstrates the versatility of the CAM system and its potential applicability to a variety of cancer types.

## Materials and reagents

**Biological materials**

1. Cell line MDA-MB-231 (ATCC^®^ HTB-26)

2. Cell line MCF-7 (ATCC^®^ HTB-22)

3. Fertilized hen eggs (*Gallus gallus*, Alpes, Puebla, México)

**Reagents**

1. Fetal bovine serum (FBS) (BIOWEST, catalog number: BIO-S1400-500); store at -20 °C; once thawed, keep at 4 °C and use within 1 month

2. DMEM/F12 (GIBCO, catalog number: 12500062 1L); store at 4 °C and use within 1 month after opening

3. Antibiotic-antimycotic 100× (AA) (BIOWEST, catalog number: BIO-L0010-100); store at -20 °C; once thawed, keep at 4 °C and use within 1 month

4. Trypsin-EDTA (0.25%) (GIBCO, catalog number: 25200072); store at 4 °C and use within 1 month after opening

5. Phosphate-buffered saline (PBS) (Fisher Scientific, catalog number: SH30256LS); store at 4 °C; once prepared, use within 1 month

6. Matrigel (CORNING, catalog number: 354234); thaw on ice and keep on ice during use; once thawed, keep at 4 °C and use within 1 month

7. Absolute ethanol (J.T. Baker, CAS No: 64-17-5); store at room temperature; stable for several years if tightly capped

8. Sodium bicarbonate (Sigma, catalog number: S5761-500G)

9. Trypan blue (HyClone, catalog number: SV30084.01)

**Solutions**

1. Supplemented culture medium (see Recipes)

2. 70% ethanol (see Recipes)

**Recipes**

**1. Supplemented culture medium**

**Table:** 

Reagent	Final concentration	Quantity or Volume
DMEM/F12	89%	89 mL
FBS	10%	10 mL
Antibiotic-antimycotic	1%	1 mL
Total	n/a	100 mL

Prepare 1,000 mL of basal DMEM/F12 culture medium according to the manufacturer’s instructions by dissolving each powder packet in 1 L of distilled water and adding 2.438 g/L of sodium bicarbonate. Sterilize the medium by filtration. The basal medium can be stored at 2–8 °C for up to four weeks. Prepare a 100 mL aliquot of supplemented medium by adding FBS and antibiotic-antimycotic and store the supplemented medium at 2–8 °C for up to four weeks.

**2. 70% ethanol**

**Table:** 

Reagent	Final concentration	Quantity or Volume
Ethanol absolute	70%	700 mL
Distilled water	30%	300 mL
Total	n/a	1,000 mL

Store at room temperature. Stable for several months in a tightly closed container.

**Laboratory supplies**

1. 100 × 20 mm cell culture plates (Santa Cruz Biotechnology, catalog number: SC251460)

2. 1.5 mL microtubes (Eppendorf, catalog number: 0030125150)

3. 10 mL serological pipettes (CORNING, catalog number: 511036)

4. 25 mL serological pipettes (CORNING, catalog number: 18293)

5. 1,000 μL pipette tips (BIOLOGIX, catalog number/SKU: 20-0200)

6. 200 μL pipette tips (BIOLOGIX, catalog number/SKU: 20-1000)

7. 15 mL conical tubes (Falcon, catalog number: 38009)

8. Style 2 precision jeweler’s tweezers, firm, non-magnetic stainless steel (MILTEX, catalog number/SKU: EF7228B)

9. Straight dissection forceps, 13 cm, stainless steel, without teeth (Hergom Medical, catalog number/SKU: 4-268-4)

10. Fine dissection scissors, straight, sharp–sharp, stainless steel, 22 mm cutting edge, 90 mm length (Fine Science Tool, catalog number/SKU: FINES01410)

11. Bottle top vacuum filter, 0.22 μm (Corning, catalog number: 430513)

## Equipment

1. Automatic egg-turning incubator (closed eggs) (CASSER, model: 200 CASSER)

2. Non-turning egg incubator (windowed eggs) (LABLINE, model: 460)

3. Laboratory centrifuge (BECKMAN, model; Spinchron Benchtop Centrifuge)

4. Water bath (Chicago Surgical & Electrical Co., model: 26100)

5. Cell culture incubator (37 °C, 5% CO_2_) (NUAIRE, model: NU-450)

6. Inverted microscope for cell culture (Olympus, model: CK2)

7. Neubauer chamber (Isolab, model: 075.03.001)

8. Class II biological safety cabinet (Forma Scientific, model: 1284)

9. Stereomicroscope (Carl Zeiss, model: Stemi 2000-C)

## Procedure

**A. Cell culture**

1. The MDA-MB-231 (1 × 10^6^) and MCF-7 (1.5 × 10^6^) cells are cultured in 100 × 20 mm plates in supplemented culture medium (see Recipes). Cultures are incubated at 37 °C in a humidified atmosphere containing 5% CO_2_. Both cell lines were obtained directly from the American Type Culture Collection (ATCC, Manassas, VA, USA), and mycoplasma testing was performed to confirm that they were negative prior to use.

*Notes:*

*1. Confirm that MCF-7 cells exhibit an epithelial morphology and that MDA-MB-231 cells display a spindle-shaped structure, with no signs of microbial contamination such as a milky appearance or turbidity of the culture medium.*

*2. A sufficient number of plates must be seeded to obtain an adequate number of cells for tumor induction. Each plate should contain approximately 2 × 10^6^ cells at 70% confluence (after 24 h for both cell lines), ensuring that cell viability was >90%.*

2. Once the chicken eggs exhibit developed vasculature, a firm chorioallantoic membrane, and reduced liquid albumen (typically on day 8 of incubation), proceed as follows: remove the culture medium and wash the cells twice with 5 mL of PBS per plate, then discard. Next, add 1 mL of trypsin and incubate at 37 °C for 5 min to facilitate cell detachment. Using an inverted microscope, verify that the cells have detached from the plate. Then, add 2 mL of supplemented culture medium.

*Notes:*

*1. Cells should reach approximately 70% confluence before proceeding with harvesting.*

*2. Repeat this procedure for each of the plates that were seeded.*

*3. A larger culture plate or flask format can be used if required.*

3. Collect the cell suspension and centrifuge at 252× *g* for 5 min to obtain a cell pellet. Resuspend the pellet in 10 mL of PBS, perform a 1:1 dilution with trypan blue, and count the cells using a Neubauer chamber.

4. Place 3 × 10^6^ cells in a tube, ensuring that cell viability is >90%, centrifuge at 252× *g* for 5 min, collect the pellet, and wash it twice with PBS. Centrifuge again, remove the PBS, and resuspend the pellet in 30 μL of Matrigel. Tubes and pipette tips should always be kept on ice to prevent premature polymerization.

5. Incubate the tube with the Matrigel–cell suspension for 15 min at 37 °C. After polymerization, the tip of a 200 μL pipette should be cut to facilitate aspiration and accurate placement of the Matrigel–cell mixture onto the egg.

**B. Preparation of embryonated eggs**

**Figure 1.**
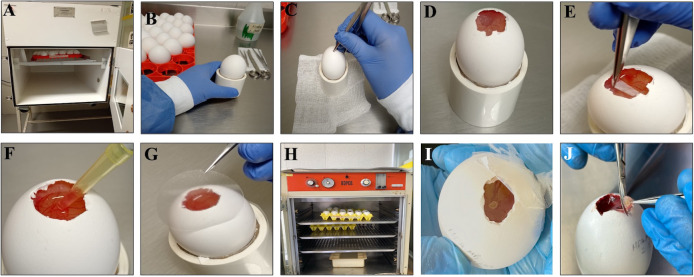
Detailed procedure for in ovo tumor induction using the chorioallantoic membrane (CAM) model. (A) Fertilized chicken eggs are incubated at 37 °C with automatic rotation and 90% relative humidity to ensure optimal embryo development. (B) On day 7 of incubation, eggs are removed from the incubator and disinfected by gently wiping the surface with gauze soaked in 70% ethanol to prevent contamination. (C–D) A window of approximately 1 cm^2^ is created at the blunt end (air sac end) by carefully removing a small section of the eggshell, exposing the external testaceous membrane. (E) The testaceous membrane is meticulously removed using fine forceps to avoid damaging the underlying vasculature, thus revealing the CAM. The window is temporarily sealed, and eggs are returned to a static incubator to maintain stable temperature and humidity until cell inoculation on day 8. (F) Tumor cells, with viability greater than 90%, are prepared in Matrigel and inoculated onto the gently lacerated CAM to enhance engraftment efficiency. (G–H) The window is then sealed with transparent tape, and the eggs are maintained in a static incubator at 37 °C with constant relative humidity (90%) to support tumor growth. (I) Tumor formation can be visually detected by stereomicroscopic inspection approximately 72 h after inoculation. (J) Tumors are harvested by excising the CAM surrounding the tumor mass with sterile scissors for downstream analyses.

1. Incubate fertilized eggs at 37 °C and 90% relative humidity in an automatic turning incubator (7 days) (Figure 1A).

*Note: Eggs should be obtained from a specialized facility that can provide a certificate confirming they are pathogen-free.*

**Critical:** Do not clean eggs with ethanol prior to incubation; this reduces embryo viability.

2. Incubate the fertilized eggs for 7 days and, after this time, remove them from the automatic turning incubator (Figure 1B).

3. Moisten a sterile gauze pad with 70% ethanol and disinfect the eggshell surface. Using forceps, create a window of approximately 1.5 cm in diameter at the blunt end of the egg (air sac end). Use precision jeweler’s tweezers to carefully remove the inner testaceous membrane and expose the chorioallantoic membrane (CAM) (Figure 1C–E).

*Note: Discard eggs in which the embryos have not developed, as well as those with malformed embryos.*

**Critical:** At this point, it is necessary to clean the eggshells with ethanol to prevent contamination after opening the egg.

4. Use transparent adhesive tape to seal the window of the eggshell, then incubate the embryos in a static incubator.

*Note: Disinfect the adhesive tape with 70% ethanol and sterilize it under UV light for 1 h prior to use. Use the tape exclusively for laboratory purposes to seal the embryos.*

**Critical:** Do not place the eggs in an automatic turning incubator at this point. Opening the egg can rupture the internal membranes and/or cause the contents to spill.

**C. Cell placement on the CAM**

1. On the day following the opening (day 8), remove the adhesive tape and verify embryo viability.

2. Place the Matrigel–cell suspension (section A) onto the CAM (Figure 1F).

*Note: The CAM should be gently lacerated using the tip of a 200 μL pipette that was previously cut with sterile scissors to create a sharp edge, in order to promote engraftment and facilitate cell deposition.*

3. Seal the eggshell window again with adhesive tape and place the eggs in a static incubator (Figure 1G).

*Note: Make sure the eggshell window is properly sealed to prevent moisture loss.*

4. Incubate the eggs inoculated with cells at 37 °C and 90% relative humidity (Figure 1H).

*Note: Throughout the incubation period, eggs should be examined daily to confirm embryo viability.*

**Critical:** In order to prevent decomposition and potential contamination of the remaining samples, dead embryos should be removed from the incubator as soon as they are identified.

5. After 72 h post–cell placement, check that tumors have formed by visual inspection using a stereomicroscope (Figure 1I).

6. Tumors can be allowed to grow for more than 3 days, depending on the experimental procedures to be performed. Once the experiment is complete, the tumor can be excised by cutting the CAM around it using scissors (Figure 1J).

## Data analysis

Tumor formation was consistently observed in >80% of inoculated embryos, and all tumors were vascularized 72 h post–cell placement.

**Figure 2.**
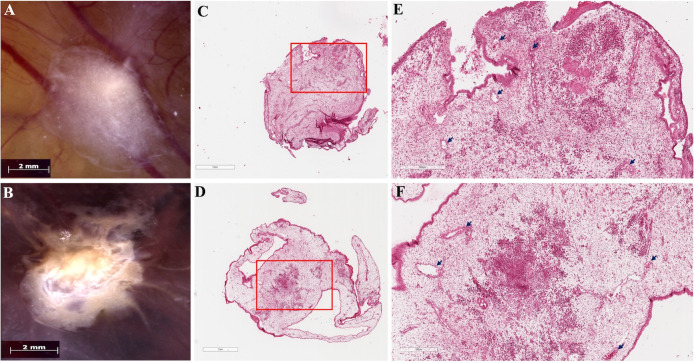
Tumors induced on the chicken embryo chorioallantoic membrane (CAM). Macroscopic images of in ovo tumors showing (A) MCF-7, characterized by a compact cellular mass with regular edges, and (B) MDA-MB-231, displaying a tumor with irregular, radially shaped edges. The vascular network of the CAM is visible beneath the tumor mass. Scale bars, 2 mm. (C–D) Panoramic views of hematoxylin-eosin-stained histological sections of the tumors developed in the CAM model: (C) MCF-7 and (D) MDA-MB-231; scale bars, 1 mm. (E–F) Higher-magnification views of the regions indicated by the red boxes in panels C and D: (E) MCF-7 and (F) MDA-MB-231. In both tumors, areas of neoplastic cells with a denser staining intensity embedded within the stroma are evident. Scale bars, 300 μm. Arrows indicate blood vessels lined by endothelial cells and containing red blood cells, present in both tumors.

We analyzed the data by comparing tumor formation rates between the experimental and control groups. Only viable embryos throughout the experiment were included in the analysis. The tumor cells can be well visualized in ovo (Figure 2A–B) and after hematoxylin-eosin staining (Figure 2C–F). As a recommendation, tumor growth may be quantitatively assessed by measuring tumor area using digital image analysis software (e.g., Digital Aperio Scanner, Leica, Wetzlar, Germany, and ImageScope software). Additionally, optional molecular characterization of CAM-induced tumors may be considered to further evaluate tumor behavior. This characterization can be performed through immunodetection approaches, including immunohistochemistry (IHC) or immunofluorescence (IF), to assess the expression of proliferation markers (e.g., Ki-67, PCNA), angiogenic markers (VEGF-A, CD31, CD34), epithelial–mesenchymal transition markers (e.g., vimentin, E-cadherin), and vasculogenic mimicry markers, such as PAS-positive/CD31-negative structures. Depending on the breast cancer cell line used and the specific study objectives, evaluation of hormone receptors and HER2 (ER/PR/HER2 status) may also be included. As an optional downstream analysis, quantitative PCR (qPCR) may be employed to evaluate early metastatic dissemination in the CAM model. This approach involves the extraction of genomic DNA from embryonic tissues (liver, lung, or CAM regions distant from the primary tumor) followed by amplification of human-specific gene sequences (such as human Alu repeats) to detect and quantify disseminated tumor cells within the chick embryo. This method provides a sensitive and quantitative assessment of tumor cell spread and has been widely used to complement histological analyses in CAM-based metastasis studies.

## Validation of protocol

Experiments were conducted with n = 30–40 embryos per group across three independent biological replicates. Negative controls included embryos inoculated with Matrigel only.

This protocol has undergone thorough testing and validation in our laboratory. It forms the experimental foundation of a completed manuscript titled “FAK Regulates Leptin-Induced Angiogenesis and Vasculogenic Mimicry in Breast Cancer” by Herrera Vargas, Ana Karen et al. (currently under review).

The protocol is an optimized version of a previous protocol, which was used and validated in the following research article: Lazzarini et al., 2016. Las nanopartículas de oro de 20 nm inhiben la proliferación e invasión de células de carcinoma mamario humano MCF-7. Mundo nano (Figures 1–3) [33]. In comparison with the protocol described by Lazzarini et al. (2016), the present study incorporated several methodological modifications. In addition to the MCF-7 cell line, the triple-negative breast cancer cell line MDA-MB-231 was also employed. Tumor cell implantation was performed on day 8 of embryo incubation, rather than at earlier stages, in order to improve embryo survival. Furthermore, the CAM was gently lacerated prior to cell inoculation to facilitate tumor engraftment. Finally, the number of tumor cells implanted per embryo was increased to enhance tumor establishment and growth.

In addition to its application in our laboratory, this protocol is supported by previously published studies in which the chicken CAM assay was successfully used for breast cancer research. Independent reports have demonstrated reliable tumor engraftment, growth, and vascularization using breast cancer cell lines: Ranjan et al., 2023. The Chorioallantoic membrane xenograft assay as a reliable model for investigating the biology of breast cancer. Cancers. (Figures 1, 3, and 4) [30]. We optimized the procedure by using a lower number of implanted cells, incorporating Matrigel to enhance tumor formation, and performing gentle laceration of the CAM to promote tumor engraftment. As a result of these modifications, tumor formation was consistently observed within a shorter experimental timeframe compared to previously reported protocols.

## General notes and troubleshooting

**General notes**

1. The experimental timeframe is limited by embryo development, restricting studies to early tumor growth.

2. The CAM supports angiogenesis and early metastasis but is not optimal for studying advanced immune responses due to the immature immune system of the embryo.

3. This model can be adapted to study drug responses or other cancer cell lines and patient-derived tumor fragments.

4. The size and vascularization of tumors may vary depending on the type of cells and the number of cells inoculated.

**Troubleshooting**

**Problem 1**: Poor embryo viability.

Possible causes: The eggs were damaged during handling, or the incubation temperature and humidity were inconsistent.

Solutions: Handle eggs gently, maintain 37 °C and 90% relative humidity, and avoid sudden movement after opening.

**Problem 2**: Low tumor take rate.

Possible causes: Insufficient number of cells, improper Matrigel preparation, or the CAM was not lacerated.

Solutions: Ensure that the number of cells inoculated is sufficient, keep Matrigel cold until use, and gently lacerate the CAM before cell application.

**Problem 3**: Hemorrhage at the tumor site.

Possible cause: Excessive CAM damage during laceration.

Solution: Make minimal and precise incisions with a cut pipette tip to avoid tearing vessels.

**Problem 4**: Contamination.

Possible cause: Nonsterile instruments or environment.

Solution: Use sterile tools and disinfect the eggshell with 70% ethanol.
